# Cost and economic burden of illness over 15 years in Nepal: A comparative analysis

**DOI:** 10.1371/journal.pone.0194564

**Published:** 2018-04-04

**Authors:** Khin Thet Swe, Md. Mizanur Rahman, Md. Shafiur Rahman, Eiko Saito, Sarah K. Abe, Stuart Gilmour, Kenji Shibuya

**Affiliations:** 1 Department of Global Health Policy, School of International Health, Graduate School of Medicine, The University of Tokyo, Japan; 2 AXA Department of Health and Human Security, Graduate School of Medicine, The University of Tokyo, Japan; Scientific Institute of Public Health (WIV-ISP), BELGIUM

## Abstract

**Background:**

With an increasing burden of non-communicable disease in Nepal and limited progress towards universal health coverage, country- and disease-specific estimates of financial hardship related to healthcare costs need to be evaluated to protect the population effectively from healthcare-related financial burden.

**Objectives:**

To estimate the cost and economic burden of illness and to assess the inequality in the financial burden due to catastrophic health expenditure from 1995 to 2010 in Nepal.

**Methods:**

This study used nationally representative Nepal Living Standards Surveys conducted in 1995 and 2010. A Bayesian two-stage hurdle model was used to estimate average cost of illness and Bayesian logistic regression models were used to estimate the disease-specific incidence of catastrophic health payment and impoverishment. The concentration curve and index were estimated by disease category to examine inequality in healthcare-related financial hardship.

**Findings:**

Inflation-adjusted mean out-of-pocket (OOP) payments for chronic illness and injury increased by 4.6% and 7.3%, respectively, while the cost of recent acute illness declined by 1.5% between 1995 and 2010. Injury showed the highest incidence of catastrophic expenditure (30.7% in 1995 and 22.4% in 2010) followed by chronic illness (12.0% in 1995 and 9.6% in 2010) and recent acute illness (21.1% in 1995 and 7.8% in 2010). Asthma, diabetes, heart conditions, malaria, jaundice and parasitic illnesses showed increased catastrophic health expenditure over time. Impoverishment due to injury declined most (by 12% change in average annual rate) followed by recent acute illness (9.7%) and chronic illness (9.6%) in 15 years. Inequality analysis indicated that poorer populations with recent acute illness suffered more catastrophic health expenditure in both sample years, while wealthier households with injury and chronic illnesses suffered more catastrophic health expenditure in 2010.

**Conclusion:**

To minimize the economic burden of illness, several approaches need to be adopted, including social health insurance complemented with an upgraded community-based health insurance system, subsidy program expansion for diseases with high economic burden and third party liability motor insurance to reduce the economic burden of injury.

## Introduction

Most low- and lower middle-income countries do not have effective financial protection schemes and rely mainly on out-of-pocket (OOP) payments for health financing.[1] The growing burden of NCDs and pre-existing communicable diseases increases the risk of high OOP payment for healthcare resulting in households being financially burdened in these countries.[2] Poor functioning health financing systems may discourage people from seeking healthcare when they need it to avoid financial burden associated with OOP payments.

To protect the population from financial catastrophe and impoverishment due to high OOP payments, Nepal has experimented with expanding insurance coverage in the past, primarily through small-scale community projects supported by NGOs and local government (Panel A in S1 Panel).[3] In addition, the government subsidizes healthcare costs for some severe illnesses for the poor population to improve access to health services and protect from financial catastrophe.[4] Despite these initiatives, the country still faces significant challenges in scaling up health insurance schemes to ensure access to health services, especially for the poor, and prevent financial burden associated with illness-related healthcare costs (Panel A in S1 Panel).[5] Although research assessing catastrophic health payment is not new in Nepal, a comprehensive overview of the economic burden of illness using nationally representative survey data across different years is still lacking (Panel B in S1 Panel). Previous studies conducted in Nepal have not clarified the amount that households spend for each illness and how much economic burden is incurred by these illnesses. Understanding the economic burden of specific illnesses is important to determine how to scale up on-going social safety net programs and disease-specific subsidy packages in Nepal.

Using 1995 and 2010 nationally representative survey data, our study aims to analyze the changes in catastrophic expenditure and impoverishment due to OOP payments over 15 years with the implementation of policies to expand programs for vulnerable populations. The specific objective of this study was to estimate the cost and economic burden of each illness including recent acute illness, chronic illness and injury as well as to assess the inequality in economic burden of illness from 1995 to 2010. This information will be important for initial steps in the implementation of financial protection systems in Nepal.

## Methods

### Data sources

Data from the Nepal Living Standards Survey in 1995 (NLSS I) and 2010 (NLSS III) were used for this study. Sample households were selected using a two-stage stratified sampling procedure in 1995 and a three-stage stratified sampling procedure in 2010 and covered the whole country, including both urban and rural areas. Under this sampling frame, 3373 households were selected in 1995 and 5988 households in 2010. The details of the sampling methods are described in the S1 Appendix (pp 2). The overall response rate was more than 99% in the both surveys. The authors had ethical approval from the University of Tokyo (certificate number: 2015–8031).

### Out-of-pocket healthcare payment

The survey collected information on OOP healthcare payment for each acute illness reported within the past 30 days and each chronic illness reported within the past year. The annual healthcare cost reported for chronic illnesses was converted into monthly cost to compare with the healthcare cost reported in the past 30 days. The questionnaires in the survey asked how much the person spent on acute illnesses in the past 30 days and on chronic illness in past 12 months. To be able to compare the economic burden of each illness, the chronic illness cost was converted into monthly cost by dividing by 12. Our assumption is that if the person has a chronic illness, they will seek health services and report OOP cost will be approximately equally distributed in each month. In this study, “chronic illness” refers to long-term suffering of illness reported within the past year of the surveys, “recent acute illness” refers to illness (other than chronic illness) reported within the past 30 days, and “injury” refers to an injury incurred in the past 30 days. The details of OOP payment variable preparation are presented in the S1 Appendix (pp 3).

### Measurement of economic burden of illness

Consistent with previous studies,[6] economic burden of illness was assessed using two financial indicators: catastrophic health expenditure and impoverishment. Healthcare expenditure is treated as catastrophic if it exceeds some fraction of total household consumption, non-food consumption, or capacity to pay.[7] In this study, we used a threshold of 10% of household total consumption, for comparability with previous studies.[8, 9] As a sensitivity analysis, we also estimated incidence of catastrophic health expenditure at alternative thresholds such as 15% of total household consumption, 40% of non-food expenditure, and 40% of capacity to pay. We estimated the economic burden of healthcare payment at two levels: household level and individual illness level. A poverty headcount was estimated using per capita total household consumption calculated with and without OOP payments for healthcare. The difference between these two poverty headcount measurements captured the impact of OOP payments on poverty. Details of the estimation procedure for catastrophic health expenditure and impoverishment are presented in the S1 Appendix (pp 3–5). Total household consumption was calculated according to the Living Standard Measurement Survey guidelines,[10] and expenditure quintile was determined using an approach provided by Xu and colleagues.[11]

### Statistical analysis

OOP health expenditure in our dataset was highly skewed due to zero costs. The stochastic process affecting participation (zero or positive expenditure) and consumption level decisions (amount of positive expenditure) may differ. To overcome this situation, a Bayesian two-stage hurdle model was used to estimate the mean OOP health expenditure.[12] A Bayesian approach addresses the concerns related to underreported illnesses or conditions. The two-stage hurdle model accounts for the both right skewed and zero-inflated nature of OOP health expenditure. The first hurdle involves the decision about whether or not to participate in healthcare expenditure due to illness (the participation decision) and was modeled with logistic regression so that it includes zero cost cases. The second hurdle concerns the level of health expenditure (the consumption decision) and models the log-transformed positive costs due to illness with linear regression. Finally, the two models were combined, with the probability of incurring a cost multiplied by expected cost to get disease-specific average health expenditure. Since prior information on the cost of each illness is difficult to obtain, we used a non-informative prior in our cost model. The Bayesian modeling code for two-stage hurdle is illustrated in the S1 Appendix (pp 7–9).

The average annual rate of change for OOP payment from 1995 to 2010 was calculated for each illness or condition, accounting for inflation using the GDP deflator obtained from the World Bank.[13] Details of the annual rate of change estimation procedure are presented in the S1 Appendix (pp 5). The average OOP healthcare cost was presented in 2010 US dollars and one US dollar was equivalent to 73.16 Rupees in 2010.

Household level catastrophic health payment and impoverishment were estimated using a Bayesian logistic regression model with informative priors. The informative prior was obtained by pooling published estimates of the incidence of household level catastrophic health payment and impoverishment in South Asia and South East Asia (Nepal, Pakistan, Bangladesh, Vietnam, India and Myanmar) by using meta-analysis. The details of the pooled prior information are shown in the Table 1. The average of sample is incorporated into our Bayesian model.

**Table 1:** Pooled prior for household level catastrophic expenditure and impoverishment.

Financial risk	Country data used	Years	Pooled incidence of catastrophic payments (95% CI)
10% total consumption	Bangladesh, India, Nepal, Pakistan, Myanmar	2010–2014	15.5 (12.3–19.0)
	Bangladesh, India, Nepal, Vietnam	1992–1996	8.9 (6.7–11.5)
25% non food	Bangladesh, India, Pakistan, Myanmar	2011–2012	17.3 (11.5–24.1)
	Bangladesh, Nepal	1992–1996	9.0 (4.5–14.8)
40% non food	Bangladesh, India, Pakistan, Myanmar	2011–2014	12.7 (8.3–17.9)
	Bangladesh, Nepal	1992–1996	4.0 (2.5–5.8)
40% capacity to pay	Bangladesh, India, Pakistan	2010–2012	5.3 (3.5–7.4)
	Bangladesh, Vietnam	1993–1995	3.8 (3.4–4.1)
Impoverishment	Bangladesh, India, Pakistan, Nepal	2011–2012	3.6 (2.9–4.4)
	Bangladesh, India, Nepal, Vietnam	1993–1996	3.1 (2.4–3.8)

Since past studies of illness-specific catastrophic expenditure and impoverishment are limited in number, prior information was difficult to obtain for some illnesses. Instead of a non-informative prior, we used a weakly informative Cauchy prior with center 0 and scale 2.5 as a default prior for our illness-specific models.[14] The posterior distribution of the Bayesian model was estimated using a Markov chain Monte Carlo (MCMC) simulation with two chains. Model robustness was confirmed with convergence diagnostics. The number of iterations was increased until the trace plots matched and the potential scale reduction factor (PSRF) was close to 1 as proposed by Gelman and colleagues.[14] All analyses were performed in JAGS and Stata/MP version 14.1.

### Confounder adjustment

We estimated the incidence of catastrophic expenditure and impoverishment for each illness model adjusted by age, sex, wealth quintiles and place of residence (rural/urban).

### Inequality assessment

To demonstrate the inequality in catastrophic healthcare payments, the concentration curve and index were estimated. The concentration curve shows the distribution of inequality in catastrophic health payment and concentration index indicates the magnitude of inequality.[7] A detailed explanation of the concentration curve and index is presented in the S1 Appendix (pp 6–7).

## Results

### Study household characteristics

Between 1995 and 2010, the proportion of all illnesses including injury and chronic illnesses increased (Table 2). Food consumption expenditure declined as a proportion of total household consumption from 53% in 1995 to 41% in 2010 while non-food consumption expenditure increased from 44% to 59% (S1 Fig).

**Table 2:** Household characteristics in Nepal, 1995–2010.

Household characteristics	Survey year	
	1995	2010
Sample size	3373	5988
Household size (mean, 95% CI)	5.7 (5.6–5.8)	4.9 (4.8–5.0)
**Age distribution, years (%, 95% CI)**		
0–4	13.1 (12.6–13.6)	10.0 (9.6–10.3)
5–19	37.4 (36.7–38.0)	36.5 (35.9–37.0)
20–59	42.9 (42.2–43.6)	44.7 (44.1–45.3)
≥ 60	6.6 (6.3–7.0)	8.8 (8.5–9.2)
**Major illness (%, 95% CI)**		
Chronic illness	29.7 (27.6–31.9)	43.0 (41.3–44.8)
Recent acute illness	41.2 (38.2–44.2)	57.0 (55.2–58.7)
Injury	2.5 (1.8–3.1)	4.5 (4.0–5.1)

95% CI: 95% confidence interval

### Frequency of illness and zero health expenditure

Incidence of each illness or condition and reported zero health expenditure are shown in Table 3. Asthma, diabetes, heart conditions, gastrointestinal disease, rheumatism and high/low blood pressure were the most commonly reported chronic illnesses in 2010. Cold/fever/flu, non-specific fever, diarrhea and respiratory illnesses were common both in 1995 and 2010. The study found a higher proportion of zero cost in more common chronic and recent acute illness categories in both years.

**Table 3:** Frequency of illness/symptom and percentage of zero health expenditure in Nepal 1995–2010.

Illness or symptom	Frequency of illness or symptom, n (%)		Percentage of zero health expenditure^a^, n (%)	
	1995	2010	1995	2010
**Chronic**				
Asthma	317 (10.6)	330 (3.8)	84 (26.5)	34 (10.3)
Diabetes	28 (0.9)	202 (2.3)	3 (10.7)	9 (4.5)
Heart conditions	134 (4.5)	187 (2.1)	34 (25.4)	25 (13.4)
Epilepsy	25 (0.8)	42 (0.5)	6 (24.0)	6 (14.3)
Occupational illness	43 (1.4)	14 (0.2)	16 (37.2)	3 (21.4)
Cancer	9 (0.3)	7 (0.1)	0	2 (28.6)
Gastrointestinal diseases	-	894 (10.2)	-	133 (14.9)
Rheumatism related	-	467 (5.3)	-	71 (15.2)
High/low blood pressure	-	400 (4.6)	-	38 (9.5)
Gynecological problems	-	140 (1.6)	-	17 (12.1)
Kidney/liver diseases	-	46 (0.5)	-	6 (13.0)
Cirrhosis of liver	87 (2.9)	-	12 (13.8)	-
**Recent acute illnesses**				
Non-specific fever	740 (24.8)	1234 (14.1)	162 (21.9)	166 (13.5)
Diarrhea	217 (7.3)	865 (9.9)	35 (16.1)	234 (27.1)
Respiratory	82 (2.8)	222 (2.5)	18 (22.0)	30 (13.5)
Skin disease	42 (1.4)	109 (1.3)	3 (7.1)	39 (35.8)
Dysentery	62 (2.1)	93 (1.1)	12 (19.4)	11 (11.8)
Malaria	34 (1.1)	71 (0.8)	11 (32.4)	15 (21.1)
Jaundice	7 (0.2)	30 (0.3)	0	2 (6.7)
Parasites	38 (1.3)	19 (0.2)	5 (13.2)	0
Measles	5 (0.2)	10 (0.1)	1 (20.0)	5 (50.0)
Tuberculosis	17 (0.6)	6 (0.1)	1 (5.9)	2 (33.3)
Cold/fever/flu	-	1713 (19.6)	-	396 (23.1)
Dental problems	-	49 (0.6)	-	11 (22.5)
**Injury**	75 (2.5)	281 (3.2)	14 (18.7)	67 (23.8)
**Other**	1018 (34.2)	1307 (15.0)	256 (25.2)	198 (15.2)
**Total**	2980 (100)	8738 (100)	668(22.4)	1520 (17.4)

^a^ individual either who did not spend any or who did not seek for treatment

### Disease-specific OOP payments

Disease-specific mean healthcare expenditure is presented in Table 4. The average annual OOP payment increased by 5% and 7% for chronic illness and injury respectively over the 15 year study period. In contrast, the average annual cost for recent acute illnesses decreased by 2%. Among chronic illnesses, asthma, diabetes, heart conditions and cancer showed significant increases in mean OOP payment over time. Among all illnesses and conditions, kidney/liver diseases, injury and heart conditions incurred the highest mean OOP health expenditure. The largest decrease was seen in tuberculosis with an average annual decrease of 12%.

**Table 4:** Disease-specific out-of-pocket healthcare payment in Nepal 1995–2010.

Illness or symptom	Mean OOP healthcare payment		Average annual rate of change, %
	(95% CrI)		
	1995*	2010*	
**Chronic**	5.9 (5.3–6.7)	11.6 (11.2–12.2)	4.6
Asthma	5.1 (4.2–6.8)	8.3 (7.9–8.5)	3.3
Diabetes	8.3 (6.5–12.0)	18.0 (17.4–18.5)	5.3
Heart conditions	11.2 (8.9–16.2)	32.4 (30.5–34.1)	7.4
Epilepsy	7.5 (4.7–13.9)	6.6 (5.7–7.3)	-0.8
Occupational illness	5.0 (2.9–10.3)	1.4 (1.0–1.7)	-8.1
Cancer	8.4 (8.4–8.4)	16.9 (8.5–22.7)	4.8
Gastrointestinal diseases	-	4.5 (4.4–4.6)	NA
Rheumatism related	-	5.5 (5.3–5.7)	NA
High/low blood pressure	-	9.3 (9.0–9.6)	NA
Gynecological problems	-	12.1 (11.3–12.8)	NA
Kidney/liver diseases	-	37.8 (33.0–41.2)	NA
Cirrhosis of liver	5.5 (4.7–7.4)	-	NA
**Recent acute illnesses**	14.3 (13.3–16.0)	11.4 (11.0–12.1)	-1.5
Non-specific fever	8.2 (7.3–9.7)	8.1 (7.7–8.9)	< -0.1
Diarrhea	9.9 (8.5–13.1)	4.5 (4.3–4.6)	-5.2
Respiratory	30.9 (22.1–59.9)	15.0 (14.2–15.7)	-4.7
Skin disease	15.4 (13.1–20.6)	4.0 (3.5–4.6)	-8.6
Dysentery	9.4 (7.2–14.8)	11.1 (10.2–11.9)	1.1
Malaria	31.8 (22.2–148.2)	12.9 (11.2–14.3)	-5.9
Jaundice	20.9 (20.9–20.9)	23.5 (20.7–25.0)	0.8
Parasites	10.2 (8.3–14.2)	17.7 (17.7–17.7)	3.8
Measles	NA	0.9 (0.4–1.4)	NA
Tuberculosis	60.3 (39.9–106.2)	8.8 (7.4–9.3)	-12.0
Cold/fever/flu	-	3.2 (3.1–3.3)	NA
Dental problems	-	7.0 (5.9–8.0)	NA
**Injury**	12.9 (10.0–20.6)	36.9 (34.5–39.3)	7.3
**Other**	13.3 (11.9–15.7)	18.4 (18.0–18.8)	2.2

95% CrI: 95% credible interval, NA: Not applicable

The average annual rate of change is estimated by converting the 1995 values to 2010 using GDP deflator. (2010 GDP deflator– 192.8, 1995 GDP deflator– 65.8)

* mean OOP in 2010 in USD, One USD was equivalent to 73.16 Rupees in 2010.

### Economic burden of illness

The incidence of catastrophic health payment and impoverishment among those who had any illness decreased by 4% and 9%, respectively between 1995 and 2010. The incidence of disease-specific catastrophic health payment in the three main disease categories decreased between the 15 years study period with an average annual decrease of 2%, 7% and 2% for chronic, recent acute illness and injury respectively (Table 5). Injury (22%) had the highest incidence of catastrophic health expenditure in both years followed by chronic illnesses (10%) and recent acute illnesses (8%) in 2010. Asthma, diabetes and heart conditions among chronic illnesses and malaria, jaundice and parasites among recent acute illnesses resulted in an increased incidence of catastrophic health payment in 15 years. About 40% of people who had cancer, kidney/liver disease and jaundice experienced financial catastrophe due to their healthcare costs in 2010. The most frequently reported recent acute illnesses (non-specific fever, diarrhea and respiratory illness) showed a considerable reduction in catastrophic health payment with an average annual decrease of 5%, 7% and 6% respectively. In 2010, around 15% (95% credible interval (CrI): 14.9–15.7) of Nepalese households faced financial catastrophe when they received healthcare services (S2 Table).

**Table 5:** Disease-specific catastrophic health payment at 10% of total consumption threshold in Nepal 1995–2010.

Illness or symptom	Incidence of catastrophic health payment (95% Credible Interval)		Average annual rate of change, %
	1995	2010	
**Chronic**	12.0 (9.6–14.6)	9.6 (8.5–10.7)	-1.5
Asthma	8.5(5.7–11.8)	11.5 (8.3–15.2)	2.0
Diabetes	7.4 (1.0–19.0)	13.9 (9.5–19.0)	4.3
Heart conditions	17.2 (11.4–24.1)	23.0 (17.2–29.3)	2.0
Epilepsy	16.2 (4.8–32.6)	12.1 (4.1–23.3)	-1.9
Occupational illness	21.0 (10.3–34.1)	NA	NA
Cancer	44.5 (16.0–75.4)	42.9 (11.9–77.2)	-0.3
Gastrointestinal diseases	-	21.3 (6.5–41.6)	NA
Rheumatism related	-	6.9 (4.7–9.3)	NA
High/low blood pressure	-	3.3 (1.8–5.2)	NA
Gynecological problems	-	16.4 (10.8–23.0)	NA
Kidney/liver diseases	-	43.5 (29.6–58.0)	NA
Cirrhosis of liver	9.2 (4.2–16.1)	-	NA
**Recent acute illnesses**	21.1 (18.9–23.4)	7.8 (7.0–8.6)	-6.4
Non-specific fever	18.9 (16.2–21.8)	9.4 (7.8–11.1)	-4.6
Diarrhea	18.9 (14.0–24.2)	6.8 (5.3–8.6)	-6.6
Respiratory	39.0 (28.7–49.9)	16.2 (11.7–21.3)	-5.7
Skin disease	33.3 (20.4–48.1)	8.3 (3.8–14.2)	-8.9
Dysentery	16.2 (8.1–26.3)	7.6 (3.1–13.7)	-4.9
Malaria	20.7 (9.1–35.6)	28.1 (18.3–39.1)	2.1
Jaundice	28.8 (4.6–63.3)	40.0 (23.7–57.7)	2.2
Parasites	13.3 (4.7–25.7)	21.3 (6.6–41.4)	3.2
Measles	20.4 (0.8–60.1)	0.7 (0.0–7.7)	-20.1
Tuberculosis	58.9 (35.2–80.0)	17.5 (0.7–52.6)	-7.8
Cold/fever/flu	-	4.3 (3.4–5.3)	NA
Dental problems	-	10.3 (3.5–20.1)	NA
**Injury**	30.7 (21.0–41.4)	22.4 (17.8–27.4)	-2.1
**Other**	20.5 (18.0–23.0)	19.8 (17.7–22.0)	-0.2
**Total**	19.3 (17.9–20.8)	10.6 (10.0–11.3)	-3.9

95% CrI: 95% credible interval, NA: Not applicable

Even though an increase in financial catastrophe was seen in some chronic and recent acute illnesses, no increase in impoverishment over these years was identified (Table 6). A considerable decline in impoverishment was seen in all three broad categories of illness; injury with an average of 12% annual decrease followed by chronic and recent acute illnesses at about 10%. The adjusted and unadjusted incidence of catastrophic health payment at different thresholds and impoverishment is presented in the supporting information (S3–S7 Tables).

**Table 6:** Disease-specific impoverishment due to healthcare payment in Nepal 1995–2010.

Illness or symptom	Incidence of impoverishment (95% CrI)		Average annual rate of change, %
	1995	2010	
**Chronic**	5.6 (4.0–7.6)	1.25 (0.9–1.7)	-9.6
Asthma	4.8 (2.8–7.4)	3.1 (1.5–5.2)	-3.0
Diabetes	3.5 (0.1–11.8)	0.04 (0.0–0.4)	-25.8
Heart conditions	8.9 (4.7–14.9)	0.6 (<0.1–2.1)	-16.8
Epilepsy	8.3 (1.2–21.1)	2.5 (0.1–8.9)	-7.7
Occupational illness	0.2 (0.0–1.9)	NA	NA
Cancer	22.4 (3.3–52.7)	1.0 (0.0–11.1)	-18.7
Gastrointestinal diseases	-	1.5 (0.8–2.3)	NA
Rheumatism related	-	1.3 (0.5–2.5)	NA
High/low blood pressure	-	0.3 (0.2–1.8)	NA
Gynecological problems	-	0.1 (0.0–1.0)	NA
Kidney/liver diseases	-	0.2 (0.0–1.6)	NA
Cirrhosis of liver	4.7 (1.3–9.8)	-	NA
**Recent acute illnesses**	8.0 (6.5–9.6)	1.7 (1.4–2.1)	-9.7
Non-specific fever	5.7 (4.1–7.5)	2.0 (1.3–2.8)	-6.9
Diarrhea	10.6 (6.8–14.9)	2.3 (1.4–3.4)	-9.6
Respiratory	11.2 (5.4–18.8)	1.8 (0.5–4.0)	-11.3
Skin disease	14.3 (6.0–25.6)	2.8 (0.6–6.6)	-10.3
Dysentery	16.3 (8.4–27.0)	1.2 (<0.1–4.1)	-16.1
Malaria	11.9 (3.4–24.1)	1.5 (0.1–5.2)	-12.9
Jaundice	14.6 (0.6–47.1)	3.6 (0.1–12.3)	-9.0
Parasites	5.3 (0.8–14.4)	5.6 (0.2–19.0)	0.4
Measles	2.3 (0.0–20.4)	0.7 (0.0–7.6)	-7.6
Tuberculosis	12.1 (1.7–31.6)	1.2 (0.0–12.8)	-14.5
Cold/fever/flu	-	1.2 (0.8–1.8)	NA
Dental problems	-	0.2 (0.0–1.6)	NA
**Injury**	14.7 (7.5–23.1)	2.2 (0.8–4.1)	-12.0
**Other**	5.2 (3.9–6.7)	1.9 (1.3–2.7)	-6.5
**Total**	6.7 (5.8–7.6)	1.6 (1.4–1.9)	-9.1

95% CrI: 95% credible interval, NA: Not applicable

### Inequality in economic burden of illness

**Fig 1:**
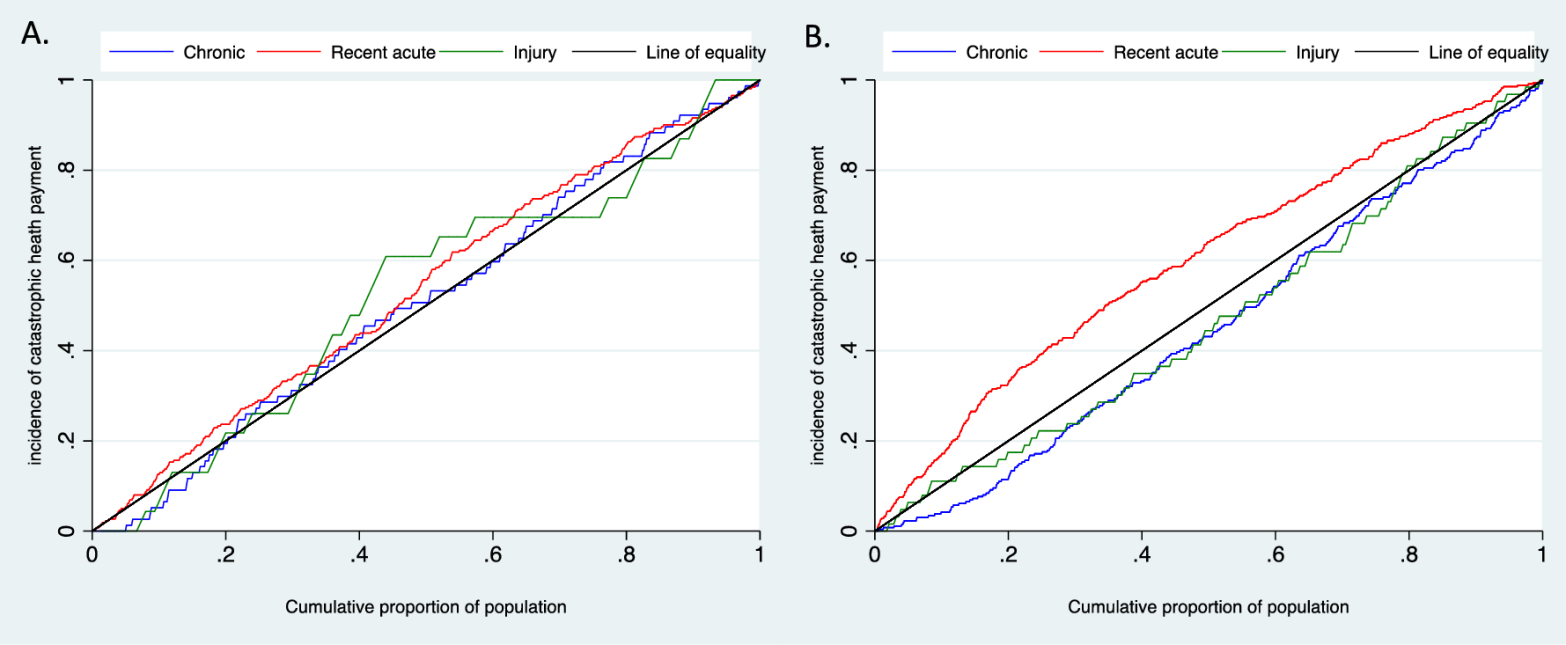
Concentration curve of catastrophic health payment by illness or symptom categories in Nepal. (A) in 1995 (B) in 2010.

**Fig 2:**
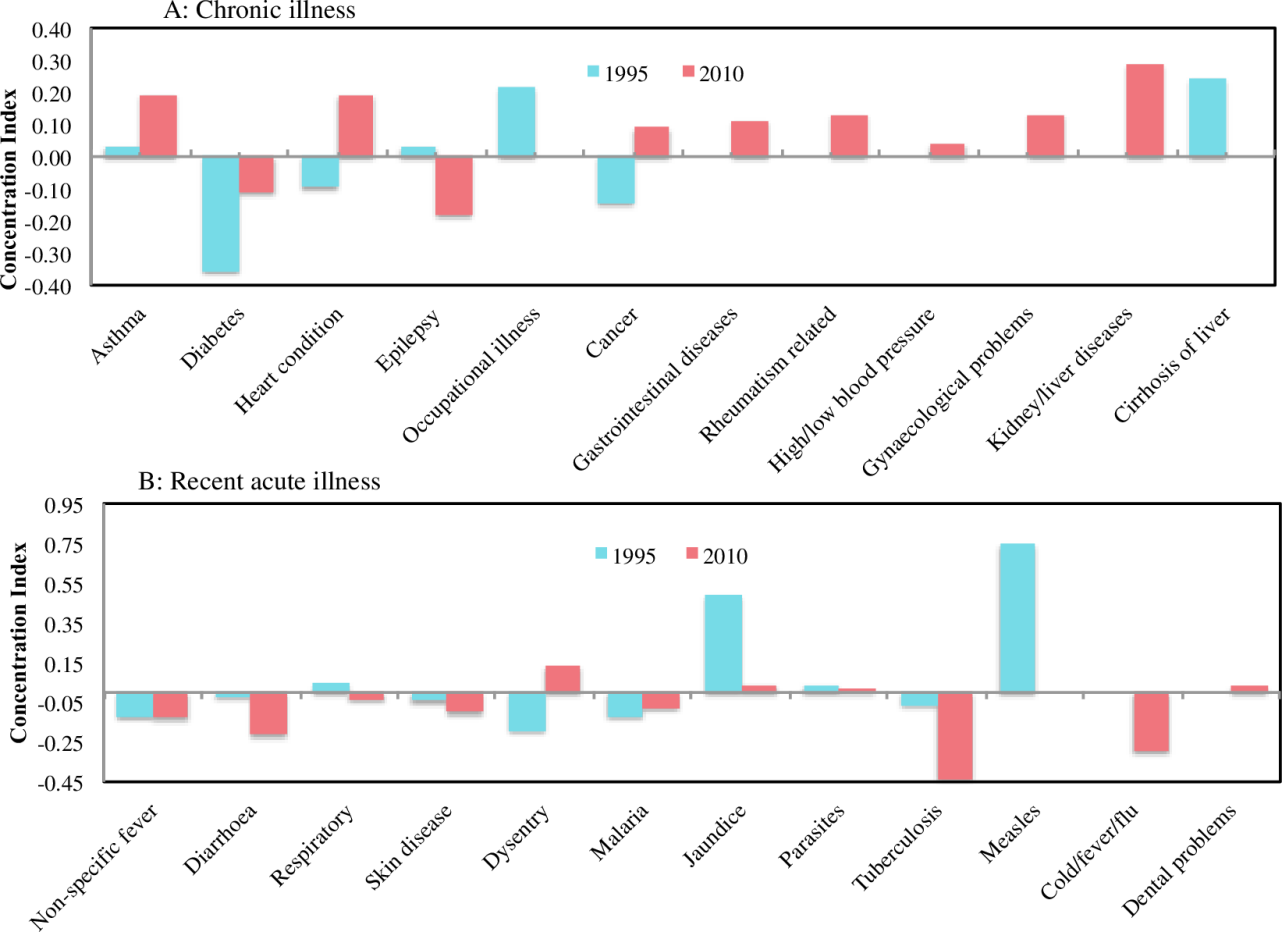
Concentration index for specific illnesses and symptoms in Nepal 1995–2010. (A) Chronic illness (B) Recent acute illness.

Fig 1 presents the concentration curves by major illness categories in the two different survey years. In 2010, the wealthy population incurred disproportionately higher catastrophic payments due to chronic illness and injury than in the poorer population; however, the results were reversed for recent acute illness. In both survey years, household catastrophic health payments were concentrated among poorer households (S2 Fig). The concentration index (CI) was high among chronic illnesses in 2010 including kidney/liver disease (CI, 0.29), asthma (CI, 0.19) and heart conditions (CI, 0.19), indicating that incidence of financial catastrophe related to these illnesses was more concentrated among the wealthy than the poor (Fig 2A). The poor population suffered more catastrophic health expenditure due to tuberculosis (CI, -0.43), cold/fever/flu (CI, -0.30) and diarrhea (CI, -0.21) than the rich in 2010 (Fig 2B).

## Discussion

Understanding household economic burden of health as well as the economic burden of specific illnesses is important to implementing universal health coverage (UHC) plans in low- and lower middle-income countries. Given the limited scope of existing studies on the economic burden due to illness in low- and lower middle-income countries, our findings provide a significant advance in understanding of the cost and economic burden of illness, including chronic illness and injury, which are of growing concern in these settings. Our findings highlight the changes in economic burden of illness over the past 15 years and the indirect contribution of implementation of various programs in Nepal. The study found average disease-specific OOP payment substantially increased in chronic illnesses and injury over 15 years; however, the economic burden of these illnesses decreased over time.

### Changes in economic burden of illness over 15 years

Consistent with a previous study,[9] around 15% of Nepalese households incurred financial catastrophe in 2010. A wide variation of financial burden was observed across illness types and over time. Increased incidence of catastrophic health expenditure over 15 years was seen in asthma, diabetes and heart conditions. Even though the economic burden caused by injury decreased, it was still one of the major illnesses associated with high economic burden. This finding is consistent with a previous study.[9] Our study found the reduction in average OOP payment and incidence of economic burden was related to the most frequently reported acute illnesses over 15 years. This reduction in economic burden related to recent acute illnesses could be due to the effect of several programs including community-based health insurance, the Free Health Service Program and the Ten-point Health Policy and Program that the government of Nepal implemented from 2003 to 2008 (Panel 1).[15] The reduction in economic burden of illnesses shows that the existing social safety net (risk protection scheme) in Nepal may help protect the population from financial burden caused by some acute illnesses.

### Illness or symptoms with high economic burden

We found high incidence of catastrophic expenditure in cancer, heart disease and kidney/liver diseases even though the government of Nepal subsidizes the treatment of these illnesses for the poor population with up to 50,000 Rupees.[4] The government of Nepal should consider proper monitoring of the subsidy program and the inclusion of chronic illness management in the current benefit packages consistent with the growing burden of chronic illness and its economic burden.[16] Injury resulted in one of the highest economic burdens in 2010. To reduce the burden related to injury, Nepal can consider injury related insurance at the time of motor vehicle purchase and implement a law related to third party liability related to fatal accidents such as those established in Brazil and India.[17]

### Changes in inequality in catastrophic payment

Despite the reduction in rates of impoverishment for all illnesses, inequality in the household economic burden of illness remained unchanged. The inequality in economic burden of illness was more profound in 2010; the poorer population suffered greater financial burden due to recent acute illness while the wealthier population suffered more due to chronic illness and injury. Even with implementation of various programs that aim to reduce the economic burden due to health payments (Panel 1), we found that poor people still suffered more economic burden due healthcare costs. The existing community-based health insurance in Nepal is also ineffective due to low population coverage, limited health service coverage, and failure to protect financial risk associated with healthcare costs.[3, 5] In contrast, countries like Germany and Japan achieved UHC through social health insurance systems which emerged from small scale community-based health insurance.[18] Therefore, Nepal could implement a social health insurance complemented by upgrading community-based health insurance to reach UHC targets by 2030.[3]

### National health spending and fiscal space

Nepal spent 5.4% of GDP on health in 2013, higher than neighboring Bangladesh and India.[19] OOP spending as a percentage of total health spending decreased from 70% in 1995 to 48% in 2014 in Nepal.[20] Increasing fiscal space for health is not feasible in this period for Nepal especially with a stagnant economy. In addition, among South Asian countries, Nepal received one of the largest amounts from donors even though dependency on external funding has been decreasing for 10 years.[21] However, mobilization of resources for health especially for prevention of life-style related illness and injury could be done through taxation on alcohol, tobacco, betel nuts and fuel.[22] Even though Nepal is in the beginning stage of tax-based financing, which is the most progressive financing source in Asia and it could be beneficial in the long term.[23]

### Strengths and limitations

Our study has several strengths. This is the first attempt to estimate the detailed economic burden of illness in Nepal using nationally representative survey data from 1995 to 2010. The Bayesian modeling analysis provides not only precise information on economic burden of specific illnesses, but also updates the incidence of catastrophic household health expenditure in Nepal by integrating previous knowledge of economic burden in a Bayesian framework. This study fills gaps in our understanding of the changes in economic burden due to healthcare costs over the past two decades and the effect of implementation of several programs to reduce health inequality. Despite these strengths, the study has a few limitations. Our study focus was assessment of the economic burden of illness. Due to the self-reported questionnaire structure, the sample size was small for some illnesses like cancer, occupational illness, measles, parasites and tuberculosis. The estimates may be slightly underestimated. However, we used Bayesian modeling that enabled us to partially handle this problem through careful choice of priors. Since the informative prior was difficult to obtain for each illness, we used a weakly informative prior for estimation of economic burden of each of the illnesses. Because transportation cost was inseparable from the data, the magnitude of OOP health expenditure is overestimated compared to other studies. However, inclusion of transportation cost in OOP health payment is important in countries like Nepal where transportation is one of the barriers to access healthcare.[24]

## Conclusion

Protection from financial catastrophe due to healthcare costs is critical in resource-limited countries like Nepal, where fiscal space is limited, dependency on OOP payments is high, coverage of risk pooling mechanisms is low, subsidy packages are not functioning effectively, diseases and economic burden related to chronic illness are increasing, and human resources for health are limited. The following approaches could enhance Nepal’s movement toward UHC and protect the population against financial hardship associated with chronic illness:

1. Social health insurance complemented with an upgraded community- based health insurance system and careful consideration of the issues caused by a fragmented health insurance scheme and benefit package;
2. Expanding the subsidy program for high economic burden especially chronic illnesses. The reimbursement should be enough to cover the economic burden caused by these diseases;
3. Third party liability motor insurance to protect the economic burden related to injury. Establish an innovative tax-based financing system together with an insurance system.

Considering the proposed approaches, carefully crafting the financing component of the health system especially in limited resource settings by prioritizing the disease burden and economic burden of the diseases in Nepal is crucial.

## Supporting information

S1 Appendix Supplementary appendix. (DOCX)

S1 Panel Panel A (Health financing reform in Nepal) and Panel B (Literature review). (DOCX)

S1 Table Disease-specific cost of illness or condition in Nepal 1995–2010. (DOCX)

S2 Table Incidence of catastrophic payment and impoverishment at the household levels in Nepal, 1995–2010. (DOCX)

S3 Table Disease-specific catastrophic health payment at 10% of total consumption threshold in Nepal 1995–2010. (DOCX)

S4 Table Disease-specific catastrophic health payment at 40% capacity to pay threshold in Nepal 1995–2010. (DOCX)

S5 Table Disease-specific catastrophic health payment at 40% non-food threshold in Nepal 1995–2010. (DOCX)

S6 Table Disease-specific catastrophic health payment at 15% total consumption threshold in Nepal 1995–2010. (DOCX)

S7 Table Disease-specific impoverishment due to healthcare payment in Nepal 1995–2010. (DOCX)

S8 Table Disease-specific concentration index in Nepal 1995–2010. (DOCX)

S1 Fig Share of household consumption in Nepal, 1995–2010. (DOCX)

S2 Fig Concentration curve of household catastrophic health payment by survey year 1995 and 2010 in Nepal. (DOCX)

S3 Fig Concentration index according to illness or symptom categories in Nepal 1995–2010. (DOCX)
