# Supplementary material for: Cost and economic burden of illness over 15 years in Nepal: A comparative analysis
Source: PLoS One. 2018 Apr 4;13(4):e0194564. doi: 10.1371/journal.pone.0194564 (PMC5884500; doi:10.1371/journal.pone.0194564)
Supplement: S1 Appendix — (DOCX) [file pone.0194564.s001.docx]

**Supplementary appendix**

Cost and economic burden of illness over 15 years in Nepal: a comparative analysis

Khin Thet Swe^1*^; Md. Mizanur Rahman^1^; Md. Shafiur Rahman^1^; Eiko Saito^2^; Sarah K. Abe^1^; Stuart Gilmour^1^ and Kenji Shibuya^1^

^1^Department of Global Health Policy, School of International Health, Graduate School of Medicine, The University of Tokyo, Japan

^2^ AXA Department of Health and Human Security, Graduate School of Medicine, The University of Tokyo, Japan

^*^ **Corresponding author**

Khin Thet Swe, M.B.B.S, MHS

Department of Global Health Policy

School of International Health

Graduate School of Medicine

The University of Tokyo

Tokyo, 113-003, Japan

E-mail: khinthetswe1989@gmail.com

**Data sources**

Nepal Living Standards Surveys in 1995 (NLSS I) and 2010 (NLSS III) were conducted by the central bureau of statistics of Nepal in cooperation with the World Bank. Sample households were selected using a two-stage stratified sampling procedure in 1995 and a three-stage stratified sampling procedure in 2010 and covered the whole country in both urban and rural areas. Under this sampling frame, 3373 households were selected in 1995 and 5988 households in 2010. In the first stage sampling (NLSS I), primary sampling units (PSU) were selected with probability proportional to size from each of the four strata. The second stage was sampled from each selected PSU with equal probabilities. In 2010, (NLSS III) consisted of two types of sample: cross-sectional sample and panel sample. The cross-sectional sample was selected by three-stage sampling procedure. The PSUs were sampled by probability proportional to size from six strata. Then, the six strata were subdivided into 14 substrata. The samples were selected from previously selected PSUs with equal probabilities. In the third stage, 12 households were equally sampled from each PSU with additional 6 PSUs, which can be used as a replacement for non-response households. The panel sample was selected from 100 PSUs in Nepal Living Standards Survey 2003 (NLSS II): 50 PSUs were selected from the cross-sectional component of NLSS II and the rest were chosen from NLSS II panel component. The details of the sampling methods are described in the Nepal Living Standards Survey report.[1, 2]

**Consumption expenditure**

The components of total household consumption aggregates included (i) food consumption, (ii) non-food consumption and (iii) durable goods. All of the components aggregated were estimated into an annual form due to the different recall period in different sections and converted into monthly estimates for incidence of catastrophic and impoverishment estimation.

The estimates of total food consumption aggregates included

1. Purchased food
2. Home production food
3. Gifted or in-kind food

The estimates of total non-food consumption aggregates included

1. Education
2. Housing and it’s utility fees
3. Personal care items
4. Household care items
5. Transportation
6. Cloths in-kind
7. Personal care in-kind
8. Household items in-kind
9. Health
10. Tobacco

The variables related to taxes, marriage, funerals and durable goods in the non-food section were excluded from our analysis.

The durable goods were estimated by using the current market value and previous value of durable goods, the age of the item from section 6 part C, the real rate of interest and rate of depreciation of durable goods according to the guideline of constructing consumption aggregates for welfare analysis by Angus Deaton and Salman Zaidi.[3] In the durable goods section in the 2010 questionnaires, the own account production of goods part from the non-food expenditure section (section 6 part D) was not included in our estimates to be consistent with the 1995 estimation.

**OOP measurements**

Out-of-pocket (OOP) health care costs reported in this study included the cost of consultation, diagnosis, medicine, transport and other expenses regardless of the place of consultation. Broad illnesses were categorized according to the illnesses listed in the 1995 and 2010 surveys. Respiratory problems were listed in both chronic and recent acute illnesses in 2010, we regarded these as recent acute illnesses to be consistent with the 1995 survey. During data management, both chronic illness and recent acute illness were combined as individual illness and the same illnesses (respiratory illness and others) that appeared twice in both categories were regarded as overlap and one was dropped from our analysis.

**Financial burden estimation**

The economic burden due to health was estimated at two levels: household level and individual illness level. The individual illness specific OOP health payment was aggregated into household level to get household OOP health payment. The household level recent acute illness OOP health payment was used to estimate our household level catastrophic health payment. If the household did not have any cost for recent acute illnesses, the OOP health payment from chronic illnesses was used for that household.

To distinguish which illnesses incur more distress to households, the incidence of disease-specific catastrophic health payment and impoverishment were estimated in each of the illnesses rather than all illnesses. Even if one household had the same type of illnesses, the cost of these illnesses was not combined to get the actual incidence of catastrophic health expenditure for each of the diseases. Our disease-specific catastrophic health expenditure may not reflect the reality of household economic burden and may be under-estimated.

OOP values in 1995 were converted into 2010 values using the GDP deflator. The GDP deflator of Nepal in 2010 was 192.8 and that of 1995 was 65.8 according to World Bank development indicators.

$${Cost}_{2010}={Cost}_{1995}*(\frac{{Deflator}_{2010}}{{Deflator}_{1995}})$$

Where,

Cost_2010_ = the value in 2010

Cost_1995_ = the value in 1995

Deflator_2010_ = GDP deflator in year 2010

Deflator_1995_ = GDP deflator in year 1995

**Measurement of catastrophic health expenditure**

The incidence of catastrophic payment is defined as exceeding a certain fraction of OOP payment as a share of total expenditure or non-food expenditure or capacity to pay. To estimate the incidence of catastrophic health expenditure, assume

- Z is set as a threshold for our fraction of catastrophic expenditure. In our paper, we chose different thresholds: more than and equal to 40% of capacity to pay, 10% or 15% of total consumption, and 40% of non food consumption.
- H is the household or individual whose OOP payment share exceeded threshold Z
- T is the OOP expenditure on health either as a household or individual illness
- X is the chosen denominator, in this case, capacity to pay, total consumption and non-food expenditure
- P is the indicator if T_i_/ X_i_ > Z equals 1 and 0 otherwise.

Our formula for incidence of catastrophic health payment is given by

$$H=\frac{1}{N}\sum_{i=1}^{N} P_{i}$$

**Measurement of impoverishment**

Impoverishment due to OOP payment is captured by estimating the difference in poverty head count with and without OOP payment

- X is the total expenditure per capita + per capita OOP health expenditure
- PL is the poverty line, in this paper, food basket poverty line
- Gross_h_ is the pre-payment poverty head count which is obtained by,

$$Gross_{h}=X <PL$$

- Net_x_ is the per capita total expenditure after paying health care
- OOP is the out-of-pocket payment for health
- Net_h_ is the post payment poverty head count which is obtained by

$$Net_{x}=X-OOP$$

$$Net_{h}=Net_{x}<PL$$

- The poverty head count due to OOP payment is estimated by

$$Diff_{h}=Net_{h}-Gross_{h}$$

For this calculation, poverty was defined as per capita total consumption expenditure less than the cost of minimum calorie requirement of 2124 kcal per day in 1995 and 2220 kcal per day in 2010. We estimated impoverishment due to OOP payment at household level and individual disease level by using the same food poverty line.

**Average annual rate of change**

In this paper, the changes in economic burden of each of illness over 15 years were estimated by using average annual growth to estimate the average change.

$$Y_{t+n}=Y_{t}{(1+b)}^{n}$$

$$\ln\left( Y_{t+n}=\ln(Y_{t} \right)+ n*\ln\left( 1+b \right)$$

$$n*\ln\left( 1+b \right)=\ln\left( Y_{t+n} \right)-\ln\left( Y_{t} \right)$$

$$\ln\left( 1+b \right)=\frac{\{\ln\left( Y_{t+n} \right)-\ln\left( Y_{t} \right)\{Saito, 2014 \#63\}}{n}$$

$$1+b=\exp\left[ \left. \frac{\left\{ \ln\left( Y_{t+n} \right)-\ln\left( Y_{t} \right) \right\}}{n} \right] \right.$$

$$b=\exp\left[ \left. \frac{\left\{ \ln\left( Y_{t+n} \right)-\ln\left( Y_{t} \right) \right\}}{n} \right] \right.-1$$

Where,

$Y_{t+n}$ = current year value

$Y_{t}$ = previous year value

*𝑏*  = average annual rate of change in percentage

n = number of year difference

**Inequality analysis**

*Concentration curve*

The concentration curve was drawn to display the degree of inequality by plotting the cumulative proportion of catastrophic health payments on the Y-axis against the cumulative proportion of population on the X-axis ranked from poorest to richest. The line of equality or the 45-degree line means an equitable distribution of catastrophic health payments among the study population irrespective of household socio-economic status. If the concentration curve lies above the line of equality, it indicates the poor population experienced more financial catastrophe compared to the rich population. If the concentration curve lies below the line of equality, it indicates the rich faced more financial catastrophe. The distance between any concentration curve and the line of equality indicates the degree of inequality, with the above (below) concentration curve showing the most pro-poor (pro-rich) distribution.

*Concentration index*

The concentration index indicates the magnitude of inequality.[5, 6] This index is directly related to the concentration curve and is defined as the area between the concentration curve and the 45-degree line (line of equality). We estimated the concentration index for survey years, chronic, recent acute illness or injury to determine magnitude of catastrophic health payment. This index indicates the extent to which the incidence of catastrophic health expenditure is concentrated among the disadvantaged or the advantaged. The concentration index gives a value ranging from -1 to 1. When the concentration index value is 0 there is no inequality i.e. catastrophic health expenditure makes no difference among the poor and rich population. A negative value indicates relatively higher catastrophic health expenditure among the poor. A positive value of the index indicates comparatively higher catastrophic health expenditure among the wealthy population.

**Programming code for Bayesian model**

*Two-stage Bayesian hurdle model to estimate the average OOP health expenditure (chronic illnesses)*

## Data loading

setwd("/bayes/baydata")

disnp<-read.table("nepal1996-2.csv",header=TRUE, sep=",")

##Keeping ng (Keeping) Variables

myvars<- c("psu", "disname", "discost","lnOOP", "coevent", "chronic1",

"age","sex","totalconsum", "resd", "hhid")

chronicdata <- disnp[myvars]

##Selecting Observations based on variable values

chronicdata <- chronicdata[which(chronicdata$chronic==1),]

attach(chronicdata)

## Mean lncost

mean(lnOOP)

## Tabulation for zero or non-zero cost

table(coevent) #shw frequency

prop.table(table(coevent)) #show percent

## Bayesian Two-stage(Hurdle) model

bugmodelchronic<-"model {

## Logistic model part

for (i in 1:n) {

coevent[i] ~ dbern(p[i])

logit(p[i])<-beta

}

## Prior

beta~dnorm(0,1.0E-10)

## Linear regression part

for (i in 1:m){

lnOOP[i]~dnorm(mu[i],tau)

mu[i]<-alpha

resid[i] <-lnOOP[i]-mu[i]

e.resid[i] <- exp(resid[i]) # Calculation of residuals

}

## Prior

alpha~dnorm(0,1.0E-6)

tau ~ dgamma(0.001,0.001)

S <- sum(e.resid[])/m # Calculation of smearing coefficients

## Predicting cost

for (i in 1:n){

cost.pred[i]<-(exp(alpha)*S)*p[i]

med.pred[i]<-(exp(alpha))*p[i]

}

## Overall mean and median cost

post_mean <- sum(cost.pred[])/n

post_med <- sum(med.pred[])/n

}"

# Data

library(rjags)

inits<-list(alpha=0,beta=0,tau=0.01)

# Data

OOP.data<-list(coevent=coevent,lnOOP=lnOOP, m=856, n=nrow(chronicdata))

jagschronic.model1<- jags.model(textConnection(bugmodelchronic),data=OOP.data, inits=inits,n.chains=2, quiet=TRUE)

update(jagschronic.model1, 100000)

chronicmodel1<- coda.samples(jagschronic.model1,

variable.names=c("post_mean","post_med"),

n.iter=100000)

summary(chronicmodel1)

traceplot(chronicmodel1)

gelman.diag(chronicmodel1)

Reference

1. Nepal living standards survey report 1995/96, Main findings, Volume one. Thapathali, Kathmandu, Nepal: National Planning Commission Secretariat, Central Bureau of Statistics, Government of Nepal; 1996.

2. Nepal living standards surveys 2010/11, Statistical Report, Volume one. Thapathali, Kathmandu, Nepal: National Planning Commission Secretariat, Central Bureau of Statistics, Government of Nepal; 2011.

3. Deaton A, Zaidi S. Guidelines for constructing consumption aggregates for welfare analysis: World Bank Publications; 2002.

4. Gelman A, Jakulin A, Pittau MG, Su Y-S. A weakly informative default prior distribution for logistic and other regression models. Ann Appl Stat. 2008:1360-83.

5. Kakwani N, Wagstaff A, Van Doorslaer E. Socioeconomic inequalities in health: measurement, computation, and statistical inference. J Econom. 1997;77(1):87-103.

6. World Health Organization. Handbook on health inequality monitoring with a special focus on low-and middle-income countries: World Health Organization; 2013.
