# Supplementary material for: Cost and economic burden of illness over 15 years in Nepal: A comparative analysis
Source: PLoS One. 2018 Apr 4;13(4):e0194564. doi: 10.1371/journal.pone.0194564 (PMC5884500; doi:10.1371/journal.pone.0194564)
Supplement: S1 Panel — (DOCX) [file pone.0194564.s002.docx]

| **Panel A: Health financing reform in Nepal** |
| --- |
| In Nepal, the percentage of general government expenditure as a percentage of total health expenditure increased from 26% in 1995 to 40% in 2014 while OOP health spending decreased from 70% to 48%.[1] The government of Nepal implemented the National Health Policy Plan in 1991. Subsequently several programs, such as the second long-term health plan and Vulnerable Community Development Plan, were implemented to increase coverage and quality of healthcare and improve access to healthcare for disadvantaged populations.[2] Six community-based health insurance schemes were established in 2003. However, they have low coverage and have failed to protect the poor population from catastrophic health costs.[3] From 2007 to 2008 the Nepalese government implemented the Free Health Service Program (for emergency and inpatient services), initially targeting the disadvantaged, which was later expanded to provide free essential healthcare services to all citizens.[4] The *Ten-point Health Policy* and Free Health Service Program offer disadvantaged populations free reproductive healthcare, especially family planning, and free essential drugs. The key target of both of these programs was to reduce the inequality in access to health services.[5, 6]  In 2013, the national health insurance policy was established as the first stage of implementation of a national health insurance system.[7] Along with this policy, the social health security scheme (SHS) was implemented with the aim of improving access to healthcare for marginalized people and people living in hard to reach areas.[7] Even though the government of Nepal has been implementing strategies for marginalized populations for over 20 years, inequality in healthcare and the effect of these programs has not yet been properly assessed. Understanding disease burden and consequential catastrophic health payments is crucial to develop an effective national health insurance benefits package. |

**Panel B: Literature review**

We searched PubMed for articles published between January 1990 and September 2016 for studies assessing the economic burden of chronic illness, acute illness and injury in South Asian countries. Keywords were “catastrophic”, “out-of-pocket”, “universal health coverage”, “impoverish”, “cost of disease”, “economic burden of disease”, “burden of illness” combined with “acute illness” OR “chronic illness” OR “injury”. The initial search identified 145 records. After title and abstract screening, 35 full-text papers were reviewed. We identified 19 papers (16 studies) reporting economic burden of illness in South Asian countries. Of the included 16 studies, eight were from India, three from Bangladesh, four from Nepal and one from Pakistan. The studies related to economic burden of injury in India, Nepal and Bangladesh reported OOP expenditure and economic loss due to injury. In Nepal, one paper reported the direct OOP cost of chronic pain and its indirect cost due to economic loss. Another study reported household catastrophic health expenditure due to some selected illnesses in Kathmandu, Nepal and focused on inequality in access to health facilities.

**Added value of this study**

Previous studies in Nepal focused on only some selected illnesses, not a comprehensive list of illnesses. The studies were limited by small sample size and performed only in urban areas and collected no data on illness-specific household spending. We estimated the economic burden of a comprehensive list of diseases using nationally representative survey data from 1995 to 2010. Cost of illness and financial burden were estimated using Bayesian modeling. To our knowledge, this is the first nationally representative study to present the economic burden of each of illness including incidence of catastrophic health expenditure and impoverishment over time in Nepal. Our study found that economic burden due to recent acute illness decreased over 15 years; however, the reduction in economic burden due to chronic illness and injury was very modest. Asthma, diabetes and heart conditions among chronic illness showed increased catastrophic expenditure over time and injury was still one of the highest burdens among all illnesses. Inequalities in household economic burden due to health are more pronounced in poorer households.

**Implications of all the available evidence**

Our study proposes that in order to increase health insurance coverage, especially among poorer people, several approaches need to be considered, including social health insurance complemented with an upgraded community-based health insurance and expanding the subsidy program for high economic burden and financially catastrophic diseases. The government of Nepal should consider the inclusion of economic burden due to chronic disease in their implementation of a social health insurance system and also implement a third party liability motor insurance system to cover the economic burden due to injury especially road traffic injury.

Reference

1. Global health expenditure database, World Health Organization, URL: http://apps.who.int/nha/database/Select/Indicators/en (accessed Aug 11, 2016).

2. The World Bank. 2016. Vulnerable community development plan. Nepal ; URL: http://documents.worldbank.org/curated/en/2016/04/26249472/nepal-health-sector-management-project-indigenous-peoples-plan-vulnerable-community-development-plan (accessed Jul 17, 2016).

3. Stoermer M, Fuerst F, Rijal K, Bhandari R, Nogier C, Gautam GS, et al. Review of community-based health insurance initiatives in Nepal. Deutsche Gesellschaft fur internationale Zusammenarbeit (GIZ) Gmbh; 2012.

4. Free health care in Nepal: Findings of a rapid assessment. Kathmandu: Health Sector Support Programme (HSSP), Ministry of Health and Population (MoHP) - Nepal, Deutsche Gesellschaft für Technische Zusammenarbeit (GTZ); 2009.

5. Prasai DP. A review of studies on Nepal's national free health care programme; Department of Health Services, Ministry of Health and Population; 2013.

6. Social management plan, Nepal health sector management project, Ministry of Health, Government of Nepal; 2016.

7. Mishra SR, Khanal P, Karki DK, Kallestrup P, Enemark U. National health insurance policy in Nepal: challenges for implementation. Glob Health Action 2015;8.
