# Supplementary material for: Cost and economic burden of illness over 15 years in Nepal: A comparative analysis
Source: PLoS One. 2018 Apr 4;13(4):e0194564. doi: 10.1371/journal.pone.0194564 (PMC5884500; doi:10.1371/journal.pone.0194564)
Supplement: S1 Table — (DOCX) [file pone.0194564.s003.docx]

S1 Table: Disease-specific cost of illness or condition in Nepal 1995-2010

| Illness or symptoms | Mean OOP healthcare payment | | | Average annual rate of change, % |
| --- | --- | --- | --- | --- |
|  | (95% CrI) | | |  |
|  | 1995 |  | 2010 |  |
| **Chronic** | 146.7 (133.4 - 166.4) |  | 849.8 (820.8 - 891.8) | 4.6 |
| Asthma | 127.3 (105.9 - 168.8) |  | 604.3 (580.8 - 624.7) | 3.3 |
| Diabetes | 206.0 (162.4 - 298.7) |  | 1318.0 (1273.0 - 1350.9) | 5.3 |
| Heart conditions | 279.4 (222.0 - 404.0) |  | 2372.4 (2229.6 - 2494.7) | 7.4 |
| Epilepsy | 186.7 (118.1 - 348.3) |  | 486.5 (418.5 - 536.0) | -0.8 |
| Occupational illness | 124.1 (73.1- 258.1) |  | 102.6 (71.1 - 124.0) | -8.1 |
| Cancer | 209.8 (209.8 - 209.8) |  | 1238.0 (622.3 - 1658.0) | 4.8 |
| Gastrointestinal diseases | - |  | 328.8 (319.5 - 337.4) | NA |
| Rheumatism related | - |  | 402.9 (386.8 - 417.7) | NA |
| High/low blood pressure | - |  | 679.5 (656.6 - 699.6) | NA |
| Gynecological problems | - |  | 885.0 (826.0 - 933.9) | NA |
| Kidney/liver diseases | - |  | 2762.3 (2414.7 - 3016.0) | NA |
| Cirrhosis of liver | 138.5 (117.0 - 184.1) |  | - | NA |
| **Recent acute illnesses** | 358.2 (332.6 - 399.3) |  | 836.6 (803.9 - 882.3) | -1.5 |
| Non-specific fever | 204.1 (181.7 - 242.9) |  | 594.6 (562.2 - 648.2) | -0.04 |
| Diarrhea | 246.1 (211.9 - 326.8) |  | 325.8 (312.5 - 338.8) | -5.2 |
| Respiratory | 770.5 (552.1 - 1494.5) |  | 1097.0 (1035.8 - 1149.2) | -4.7 |
| Skin disease | 383.9 (328.0 - 514.0) |  | 294.4 (252.6 - 334.2) | -8.6 |
| Dysentery | 235.5 (180.5 - 369.5) |  | 814.7 (747.7 - 867.5) | 1.1 |
| Malaria | 794.6 (554.6 - 3700.3)* |  | 943.1 (821.9 - 1045.7) | -5.9 |
| Jaundice | 522.6 (522.6 - 522.6) |  | 1722.0 (1516.8 - 1829.0) | 0.8 |
| Parasites | 254.7 (206.4 - 355.5) |  | 1298.2 (1298.0 - 1298.2) | 3.8 |
| Measles | NA |  | 65.2 (27.6 - 102.7) | NA |
| Tuberculosis | 1506.6 (995.2 - 2652.0) |  | 644.0 (544.5 - 683.9) | -12.0 |
| Cold/fever/flu | - |  | 232.8 (226.7 - 238.8) | NA |
| Dental problems | - |  | 515.7 (430.8 - 585.1) | NA |
| **Injury** | 322.0 (248.6 - 513.9) |  | 2702.8 (2520.9 - 2872.0) | 7.3 |
| **Other** | 331.3 (296.8 - 391.7) |  | 1347.5 (1315.8 - 1377.6) | 2.2 |

95% CrI: 95% credible interval, NA: Not applicable.

Mean OOP healthcare payment in 1995 and 2010 local currency.

The average annual rate of change is estimated by converting the 1995 values to 2010 using GDP deflator. (2010 GDP deflator – 192.8, 1995 GDP deflator – 65.8)

* The median of the out-of-pocket payment
