# Supplementary material for: Cost and economic burden of illness over 15 years in Nepal: A comparative analysis
Source: PLoS One. 2018 Apr 4;13(4):e0194564. doi: 10.1371/journal.pone.0194564 (PMC5884500; doi:10.1371/journal.pone.0194564)
Supplement: S2 Table — (DOCX) [file pone.0194564.s004.docx]

S2 Table: Incidence of catastrophic payment and impoverishment at the household levels in Nepal, 1995 – 2010

| Financial risk | Incidence of catastrophic payments  (95% CrI) | |
| --- | --- | --- |
|  | 1995 | 2010 |
| Total household consumption |  |  |
| >10% | 9.5 (9.2 - 9.7) | 15.3 (14.9 - 15.7) |
| Non-food consumption |  |  |
| >25% | 13.2 (12.5 - 13.9) | 17.0 (16.6 - 17.4) |
| >40% | 8.0 (7.4 - 8.6) | 12.2 (11.8 - 12.5) |
| Capacity to pay |  |  |
| ≥40% | 7.1 (6.6 - 7.7) | 9.6 (9.3 - 9.8) |
| Impoverishment | 3.0 (2.8 - 3.1) | 3.0 (2.8 - 3.2) |

95% CrI: 95% credible interval
