# Supplementary material for: Cost and economic burden of illness over 15 years in Nepal: A comparative analysis
Source: PLoS One. 2018 Apr 4;13(4):e0194564. doi: 10.1371/journal.pone.0194564 (PMC5884500; doi:10.1371/journal.pone.0194564)
Supplement: S3 Table — (DOCX) [file pone.0194564.s005.docx]

S3 Table: Disease-specific catastrophic health payment at 10% of total consumption threshold in Nepal 1995 - 2010

| Illness or symptoms | Multivariable adjusted | | |
| --- | --- | --- | --- |
|  | Incidence of catastrophic health payment (95% CrI) | | |
|  | 1995 |  | 2010 |
| **Chronic** | 12.0 (9.6 - 14.6) |  | 9.6 (8.5 - 10.7) |
| Asthma | 8.5 (5.7 - 11.8) |  | 11.5 (8.3 - 15.1) |
| Diabetes | 7.4 (1.6 - 15.7) |  | 13.9 (9.6 - 18.6) |
| Heart conditions | 17.2 (11.5 - 23.6) |  | 23.1 (17.5 - 29.1) |
| Epilepsy | 16.1 (5.4 - 30.6) |  | 12.0 (4.5 - 21.8) |
| Occupational illnesses | 21.1 (11.4 - 32.7) |  | 0.1 (0.0 - 0.1) |
| Cancer | NA* |  | 43.2 (25.0 - 62.6) |
| Gastrointestinal problems | - |  | 21.2 (7.5 - 38.7) |
| Rheumatism related | - |  | 6.9 (4.8 - 9.3) |
| High/low blood pressure | - |  | 3.3 (1.8 - 5.2) |
| Gynecological problems | - |  | 16.4 (10.8 - 22.9) |
| Kidney/liver diseases | - |  | 43.4 (30.9 - 56.3) |
| Cirrhosis of liver | 9.3 (4.4 - 15.5) |  | - |
| **Recent acute illnesses** | 21.1 (18.9 - 23.3) |  | 7.8 (7.0 - 8.6) |
| Non-specific fever | 18.9 (16.2 - 21.8) |  | 9.4 (7.9 - 11.0) |
| Diarrhea | 18.9 (14.1 - 24.3) |  | 6.8 (5.3 - 8.5) |
| Respiratory | 39.0 (29.5 - 49.1) |  | 16.2 (11.7 - 21.3) |
| Skin Disease | 33.2 (21.0 - 46.5) |  | 8.3 (4.0 - 14.0) |
| Dysentery | 16.2 (8.6 - 25.4) |  | 7.6 (3.2 - 13.3) |
| Malaria | 20.9 (10.3 - 33.3) |  | 28.3 (19.6 - 37.9) |
| Jaundice | NA* |  | 40.0 (25.5 - 55.7) |
| Parasites | 13.2 (6.4 - 21.5) |  | 21.4 (7.6 - 38.8) |
| Measles | NA* |  | <0.1 (0.0 - 0.01) |
| Tuberculosis | 59.1 (39.3 - 77.7) |  | NA* |
| Cold/fever/flu | - |  | 4.3 (3.4 - 5.3) |
| Dental Problems | - |  | 10.3 (3.7 - 19.3) |
| **Injury** | 30.7 (21.7 - 40.4) |  | 22.5 (17.8 - 27.5) |
| **Other** | 20.4 (18.0 - 22.9) |  | 19.9 (17.7 – 22.0) |

95% CrI: 95% credible interval, NA: Not applicable

* The model cannot be further assessed due to the limited sample size
