# Supplementary material for: Cost and economic burden of illness over 15 years in Nepal: A comparative analysis
Source: PLoS One. 2018 Apr 4;13(4):e0194564. doi: 10.1371/journal.pone.0194564 (PMC5884500; doi:10.1371/journal.pone.0194564)
Supplement: S4 Table — (DOCX) [file pone.0194564.s006.docx]

S4 Table: Disease-specific catastrophic health payment at 40% capacity to pay threshold in Nepal 1995 - 2010

| Illness or symptoms | Incidence of catastrophic health payment (95% CrI) | | | | | | | |
| --- | --- | --- | --- | --- | --- | --- | --- | --- |
|  | Unadjusted model | | | | Multivariable adjusted model | | | |
|  | 1995 |  | 2010 |  | | 1995 |  | 2010 |
| **Chronic** | 5.6 (4.0 - 7.5) |  | 3.4 (2.8 - 4.1) |  | | 5.6 (4.0 - 7.5) |  | 3.4 (2.8 - 4.1) |
| Asthma | 4.1 (2.2 - 6.5) |  | 3.7 (1.9 - 5.9) |  | | 4.1 (2.3 - 6.5) |  | 3.7 (1.9 - 5.9) |
| Diabetes | 3.8 (0.1 - 13.0) |  | 3.0 (1.1 - 5.8) |  | | 3.7 (0.3 - 8.6) |  | 3.0 (1.2 - 5.6) |
| Heart conditions | 8.2 (4.2 - 13.4) |  | 9.1 (5.5 - 13.7) |  | | 8.3 (4.3 - 13.3) |  | 9.1 (5.6 - 13.4) |
| Epilepsy | 8.2 (1.1 - 21.6) |  | 7.2 (1.6 - 16.4) |  | | 8.2 (1.6 - 18.3) |  | 7.2 (1.8 - 15.4) |
| Occupational illness | 11.7 (4.2 - 22.6) |  | 0.5 (0.0 - 5.5) |  | | 11.8 (4.7 - 21.3) |  | 0.1 (0.0 - 0.1) |
| Cancer | 11.8 (0.5 - 38.1) |  | 15.0 (0.6 - 46.8) |  | | 11.7 (1.7 - 23.5) |  | NA* |
| Gastrointestinal diseases | - |  | 2.1 (1.3 - 3.2) |  | | - |  | 2.1 (1.3 - 3.2) |
| Rheumatism related | - |  | 2.2 (1.0 - 3.7) |  | | - |  | 2.2 (1.1 - 3.6) |
| High/low blood pressure | - |  | 0.8 (0.2 - 1.8) |  | | - |  | 0.8 (0.2 - 1.8) |
| Gynecological problems | - |  | 11.5 (6.7 - 17.3) |  | | - |  | 11.5 (6.9 - 17.1) |
| Kidney/liver diseases | - |  | 13.1 (5.0 - 24.1) |  | | - |  | 13.2 (6.5 - 22.0) |
| Cirrhosis of liver | 3.5 (0.7 - 8.1) |  | - |  | | 3.6 (0.9 - 7.6) |  | - |
| **Recent acute illnesses** | 17.7 (15.6 - 19.9) |  | 5.0 (4.4 - 5.7) |  | | 17.7 (15.7 - 19.8) |  | 5.0 (4.4 - 5.7) |
| Non-specific fever | 16.5 (13.9 - 19.3) |  | 5.7 (4.5 - 7.0) |  | | 16.5 (14.0 - 19.1) |  | 5.7 (4.5 - 7.0) |
| Diarrhea | 14.7 (10.3 - 19.7) |  | 5.6 (4.1 - 7.2) |  | | 14.8 (10.5 - 19.4) |  | 5.6 (4.1 - 7.1) |
| Respiratory | 34.2 (24.3 - 44.8) |  | 12.2 (8.3 - 16.7) |  | | 34.1 (24.6 - 44.1) |  | 12.2 (8.3 - 16.6) |
| Skin disease | 21.5 (10.6 - 34.8) |  | 5.6 (2.1 - 10.5) |  | | 21.5 (11.4 - 33.4) |  | 5.6 (2.3 - 9.9) |
| Dysentery | 11.4 (4.8 - 20.3) |  | 6.6 (2.5 - 12.4) |  | | 11.5 (5.1 - 19.5) |  | 6.5 (2.6 - 11.8) |
| Malaria | 20.7 (9.0 - 35.6) |  | 15.5 (8.2 - 24.8) |  | | 20.7 (10.2 - 33.2) |  | 15.5 (8.6 - 23.8) |
| Jaundice | 29.1 (4.7 - 64.9) |  | 16.8 (5.9 - 31.7) |  | | NA* |  | 16.8 (6.7 - 29.6) |
| Parasites | 10.7 (3.1 - 21.9) |  | 16.0 (3.6 - 35.5) |  | | 10.5 (3.9 - 19.6) |  | 16.1 (4.4 - 32.2) |
| Measles | 1.4 (0.0 - 16.1) |  | 0.7 (0.0 - 7.6) |  | | NA* |  | NA* |
| Tuberculosis | 52.8 (30.1 - 75.2) |  | 17.5 (0.7 - 53.2) |  | | 53.4 (33.8 - 72.3) |  | NA* |
| Cold/fever/flu | - |  | 2.5 (1.8 - 3.3) |  | | - |  | 2.5 (1.9 - 3.3) |
| Dental problems | - |  | 4.2 (0.6 - 11.2) |  | | - |  | 4.2 (6.2 - 10.6) |
| **Injury** | 29.4 (19.8 - 40.2) |  | 14.2 (10.4 - 18.5) |  | | 29.3 (20.4 - 39.2) |  | 14.3 (10.5 - 18.5) |
| **Other** | 14.8 (12.7 - 17.0) |  | 12.2 (10.5 - 14.0) |  | | 14.7 (12.7 - 16.9) |  | 12.2 (10.5 - 14.0) |

95% CrI: 95% credible interval, NA: Not applicable

* The model cannot be further assessed due to the limited sample size
