# Supplementary material for: Cost and economic burden of illness over 15 years in Nepal: A comparative analysis
Source: PLoS One. 2018 Apr 4;13(4):e0194564. doi: 10.1371/journal.pone.0194564 (PMC5884500; doi:10.1371/journal.pone.0194564)
Supplement: S5 Table — (DOCX) [file pone.0194564.s007.docx]

S5 Table: Disease-specific catastrophic health payment at 40% non-food threshold in Nepal 1995 - 2010

| Illness or symptoms | Incidence of catastrophic health payment (95% CrI) | | | | | | | |
| --- | --- | --- | --- | --- | --- | --- | --- | --- |
|  | Unadjusted model | | | | Multivariable adjusted model | | | |
|  | 1995 |  | 2010 |  | | 1995 |  | 2010 |
| **Chronic** | 7.3 (5.4 - 9.4) |  | 4.1 (3.4 - 4.8) |  | | 7.3 (5.5 - 9.4) |  | 4.1 (3.4 - 4.8) |
| Asthma | 4.4 (2.5 - 6.9) |  | 4.0 (2.1 - 6.3) |  | | 4.4 (2.5 - 6.9) |  | 4.0 (2.2 - 6.3) |
| Diabetes | 7.2 (1.0 - 18.5) |  | 5.0 (2.4 - 8.4) |  | | 7.2 (1.3 - 15.9) |  | 5.0 (2.5 - 8.2) |
| Heart conditions | 11.2 (6.5 - 17.0) |  | 10.2 (6.2 - 14.9) |  | | 11.2 (6.6 - 16.9) |  | 10.2 (6.4 - 14.6) |
| Epilepsy | 12.1 (2.6 - 27.1) |  | 7.2 (1.6 - 16.4) |  | | 12.2 (3.5 - 24.9) |  | 7.3 (1.8 - 15.6) |
| Occupational illness | 16.4 (7.1 - 28.7) |  | 0.5 (0.0 - 5.5) |  | | 16.3 (7.8 - 27.1) |  | 0.1 (0.0 - 0.1) |
| Cancer | 22.4 (3.2 - 52.9) |  | 28.6 (4.3 - 64.4) |  | | 23.4 (9.3 - 38.4) |  | NA* |
| Gastrointestinal diseases | - |  | 2.6 (1.7 - 3.7) |  | | - |  | 2.6 (1.7 - 3.7) |
| Rheumatism related | - |  | 2.6 (1.3 - 4.2) |  | | - |  | 2.6 (1.4 - 4.1) |
| High/low blood pressure | - |  | 1.5 (0.6 - 2.9) |  | | - |  | 1.5 (0.6 - 2.9) |
| Gynecological problems | - |  | 11.5 (6.7 - 17.3) |  | | - |  | 11.5 (6.8 - 17.0) |
| Kidney/liver diseases | - |  | 15.3 (6.6 - 26.7) |  | | - |  | 15.3 (7.8 - 24.9) |
| Cirrhosis of liver | 4.6 (1.3 - 9.9) |  | - |  | | 4.7 (1.5 - 9.3) |  | - |
| **Recent acute illnesses** | 20.6 (18.3 - 22.9) |  | 5.4 (4.7 - 6.1) |  | | 20.6 (18.4 - 22.8) |  | 5.4 (4.8 - 6.1) |
| Non-specific fever | 19.4 (16.7 - 22.4) |  | 6.2 (4.9 - 7.6) |  | | 19.5 (16.7 - 22.4) |  | 6.2 (4.9 - 7.5) |
| Diarrhea | 17.5 (12.8 - 22.9) |  | 5.8 (4.3 - 7.5) |  | | 17.5 (12.9 - 22.6) |  | 5.8 (4.4 - 7.4) |
| Respiratory | 34.2 (24.5 - 44.7) |  | 13.6 (9.4 - 18.3) |  | | 34.2 (24.8 - 44.0) |  | 13.6 (9.5 - 18.2) |
| Skin disease | 26.2 (14.2 - 40.3) |  | 5.6 (2.1 - 10.6) |  | | 26.2 (14.8 - 38.9) |  | 5.6 (2.3 - 9.9) |
| Dysentery | 12.9 (5.7 - 22.3) |  | 7.5 (3.1 - 13.6) |  | | 13.0 (6.3 - 21.1) |  | 7.6 (3.3 - 13.4) |
| Malaria | 20.6 (8.9 - 35.8) |  | 16.9 (9.3 - 26.3) |  | | 20.8 (10.4 - 32.9) |  | 17.0 (9.8 - 25.4) |
| Jaundice | 28.8 (4.5 - 64.2) |  | 16.8 (5.9 - 32.2) |  | | NA* |  | 16.7 (6.8 - 28.9) |
| Parasites | 18.4 (7.9 - 32.1) |  | 16.0 (3.8 - 34.8) |  | | 18.4 (9.6 - 29.3) |  | 16.1 (4.7 - 31.8) |
| Measles | 20.2 (0.8 - 59.5) |  | 0.7 (0.0 - 7.6) |  | | NA* |  | NA* |
| Tuberculosis | 58.7 (35.3 - 80.1) |  | 17.2 (0.7 - 52.4) |  | | 59.1 (39.6 - 76.8) |  | NA* |
| Cold/fever/flu | - |  | 2.6 (1.9 - 3.4) |  | | - |  | 2.6 (1.9 - 3.4) |
| Dental problems | - |  | 6.2 (1.4 - 14.3) |  | | - |  | 6.3 (1.5 - 13.6) |
| **Injury** | 30.6 (20.7 - 41.5) |  | 15.0 (11.1 - 19.3) |  | | 30.7 (21.6 - 40.6) |  | 14.9 (11.1 - 19.3) |
| **Other** | 17.1 (14.8 - 19.5) |  | 13.5 (11.8 - 15.5) |  | | 17.1 (14.9 - 19.4) |  | 13.5 (11.7 - 15.4) |

95% CrI: 95% credible interval, NA: Not applicable

* The model cannot be further assessed due to the limited sample size
