# Supplementary material for: Cost and economic burden of illness over 15 years in Nepal: A comparative analysis
Source: PLoS One. 2018 Apr 4;13(4):e0194564. doi: 10.1371/journal.pone.0194564 (PMC5884500; doi:10.1371/journal.pone.0194564)
Supplement: S6 Table — (DOCX) [file pone.0194564.s008.docx]

S6 Table: Disease-specific catastrophic health payment at 15% total consumption threshold in Nepal 1995 - 2010

| Illness or symptoms | Incidence of catastrophic health payment (95% CrI) | | | | | | | |
| --- | --- | --- | --- | --- | --- | --- | --- | --- |
|  | Unadjusted model | | | | Multivariable adjusted model | | | |
|  | 1995 |  | 2010 |  | | 1995 |  | 2010 |
| **Chronic** | 5.9 (4.2 - 7.9) |  | 5.7 (4.8 - 6.6) |  | | 5.9 (4.3 - 7.8) |  | 5.7 (4.9 - 6.6) |
| Asthma | 3.2 (1.5 - 5.3) |  | 6.7 (4.2 - 9.6) |  | | 3.2 (1.6 - 5.3) |  | 6.7 (4.3 - 9.6) |
| Diabetes | 0.4 (0.0 - 3.5) |  | 7.5 (4.3 - 11.4) |  | | 0.3 (0.0 - 3.0) |  | 7.5 (4.3 - 11.2) |
| Heart conditions | 9.7 (5.4 - 15.2) |  | 12.3 (8.0 - 17.3) |  | | 9.7 (5.5 - 14.9) |  | 12.3 (8.2 - 17.0) |
| Epilepsy | 12.1 (2.8 - 26.9) |  | 9.6 (2.9 - 19.9) |  | | 12.2 (3.4 - 24.8) |  | 9.7 (3.1 - 18.7) |
| Occupational illness | 14.0 (5.6 - 25.9) |  | 0.5 (0.0 - 5.5) |  | | 14.1 (6.2 - 24.1) |  | 0.1 (0.0 - 0.2) |
| Cancer | 11.4 (0.3 - 37.5) |  | 43.2 (12.2 - 78.0) |  | | 12.7 (1.4 - 26.8) |  | NA* |
| Gastrointestinal diseases | - |  | 3.1 (1.6 - 4.6) |  | | - |  | 3.0 (2.0 - 4.2) |
| Rheumatism related | - |  | 4.1 (2.5 - 6.1) |  | | - |  | 4.1 (2.5 - 6.0) |
| High/low blood pressure | - |  | 2.0 (0.8 - 3.6) |  | | - |  | 2.0 (0.9 - 3.5) |
| Gynecological problems | - |  | 13.6 (8.5 - 19.6) |  | | - |  | 13.5 (8.5 - 19.5) |
| Kidney/liver diseases | - |  | 32.7 (20.1 - 46.8) |  | | - |  | 32.6 (21.3 - 45.0) |
| Cirrhosis of liver | 5.8 (2.0 - 11.5) |  | - |  | | 5.9 (2.4 - 10.4) |  | - |
| **Recent acute illnesses** | 13.4 (11.6 - 15.4) |  | 4.7 (4.1 - 5.3) |  | | 13.4 (11.6 - 15.4) |  | 4.7 (4.1 - 5.3) |
| Non-specific fever | 11.6 (9.4 - 14.0) |  | 5.3 (4.1 - 6.6) |  | | 11.6 (9.5 - 14.0) |  | 5.3 (4.1 - 6.6) |
| Diarrhea | 12.0 (8.1 - 16.7) |  | 5.0 (3.6 - 6.5) |  | | 12.0 (8.2 - 16.4) |  | 5.0 (3.7 - 6.5) |
| Respiratory | 30.5 (21.1 - 40.8) |  | 13.5 (9.4 - 18.3) |  | | 30.5 (21.5 - 40.4) |  | 13.1 (9.0 - 17.7) |
| Skin disease | 19.2 (8.8 - 32.1) |  | 3.7 (1.0 - 8.1) |  | | 19.1 (9.2 - 31.3) |  | 3.8 (1.1 - 7.7) |
| Dysentery | 8.1 (2.8 - 16.0) |  | 6.5 (2.5 - 12.4) |  | | 8.3 (3.1 - 15.2) |  | 6.5 (2.5 - 11.8) |
| Malaria | 14.8 (5.3 - 28.3) |  | 14.1 (7.2 - 22.9) |  | | 14.9 (6.5 - 25.5) |  | 14.2 (7.3 - 22.4) |
| Jaundice | 14.6 (0.5 - 46.1) |  | 20.0 (8.0 - 35.8) |  | | NA* |  | 20.0 (9.0 - 33.7) |
| Parasites | 7.9 (1.7 - 18.2) |  | 16.0 (3.5 - 34.8) |  | | 7.8 (2.7 - 14.4) |  | 16.0 (4.5 - 32.0) |
| Measles | 1.8 (0.0 - 17.7) |  | 0.7 (0.0 - 7.6) |  | | 0.1 (0.0 - 0.1) |  | NA* |
| Tuberculosis | 47.1 (24.7 - 69.7) |  | 17.2 (0.7 - 52.4) |  | | 47.4 (29.0 - 66.0) |  | NA* |
| Cold/fever/flu | - |  | 2.3 (1.6 - 3.0) |  | | - |  | 2.2 (1.6 - 3.0) |
| Dental problems | - |  | 4.2 (0.5 - 11.2) |  | | - |  | 4.2 (0.6 - 10.3) |
| **Injury** | 29.3 (19.6 - 40.1) |  | 15.7 (11.7 - 20.1) |  | | 29.4 (20.5 - 39.0) |  | 15.7 (11.7 - 20.1) |
| **Other** | 14.2 (12.1 - 16.4) |  | 14.9 (13.1 - 16.9) |  | | 14.0 (11.9 - 16.1) |  | 15.0 (13.1 - 17.0) |

95% CrI: 95% credible interval, NA: Not applicable

* The model cannot be further assessed due to the limited sample size
