# Supplementary material for: Cost and economic burden of illness over 15 years in Nepal: A comparative analysis
Source: PLoS One. 2018 Apr 4;13(4):e0194564. doi: 10.1371/journal.pone.0194564 (PMC5884500; doi:10.1371/journal.pone.0194564)
Supplement: S7 Table — (DOCX) [file pone.0194564.s009.docx]

S7 Table: Disease-specific impoverishment due to healthcare payment in Nepal 1995 - 2010

| Illness or symptoms | Multivariable adjusted model | |
| --- | --- | --- |
|  | Incidence of impoverishment (95% CrI) | |
|  | 1995 | 2010 |
| **Chronic** | 5.6 (4.0 - 7.4) | 1.3 (0.9 - 1.7) |
| Asthma | 4.7 (2.8 - 7.3) | 3.1 (1.6 - 4.9) |
| Diabetes | NA* | NA* |
| Heart conditions | 9.0 (5.2 - 13.9) | 0.6 (<0.1 - 1.8) |
| Epilepsy | 8.1 (3.9 - 14.1) | 2.4 (0.4 - 4.7) |
| Occupational illnesses | 0.2 (0.0 - 2.1) | 0.1 (0.0 - 0.2) |
| Cancer | 24.1 (10.0 - 39.4) | 0.1 (0.0 - 0.8) |
| Gastrointestinal problems | - | 1.5 (0.8 - 2.3) |
| Rheumatism related | - | 1.3 (0.5 - 2.4) |
| High/low blood pressure | - | 0.3 (<0.1 - 0.9) |
| Gynecological problems | - | 1.5 (0.2 - 3.7) |
| Kidney/liver diseases | - | NA* |
| Cirrhosis of liver | 4.7 (1.4 - 9.5) | - |
| **Recent acute illnesses** | 8.0 (6.6 - 9.4) | 1.7 (1.4 - 2.1) |
| Non-specific fever | 5.7 (4.2 - 7.5) | 2.0 (1.3 - 2.8) |
| Diarrhea | 10.6 (7.0 - 14.8) | 2.3 (1.5 - 3.4) |
| Respiratory | 11.1 (5.7 - 17.7) | 1.8 (0.6 - 3.7) |
| Skin Disease | 14.3 (6.8 - 23.7) | 2.8 (0.8 - 5.5) |
| Dysentery | 16.2 (9.3 - 23.7) | 1.1 (0.2 - 2.0) |
| Malaria | 12.1 (6.0 - 18.7) | 1.5 (0.1 - 3.5) |
| Jaundice | 15.6 (1.9 - 31.9) | 3.4 (0.8 - 5.9) |
| Parasites | 5.5 (0.8 - 13.6) | 5.3 (2.6 - 8.2) |
| Measles | NA* | NA* |
| Tuberculosis | 11.8 (2.3 - 26.9) | NA* |
| Cold/fever/flu | - | 1.2 (0.8 - 1.8) |
| Dental Problems | - | NA* |
| **Injury** | 14.8 (8.0 - 22.8) | 2.2 (0.8 - 4.1) |
| **Other** | 5.2 (3.9 - 6.6) | 1.9 (1.3 - 2.7) |

95% CrI: 95% credible interval, NA: Not applicable

* The model cannot be further assessed due to the limited sample size.
