# Supplementary material for: Cost and economic burden of illness over 15 years in Nepal: A comparative analysis
Source: PLoS One. 2018 Apr 4;13(4):e0194564. doi: 10.1371/journal.pone.0194564 (PMC5884500; doi:10.1371/journal.pone.0194564)
Supplement: S8 Table — (DOCX) [file pone.0194564.s010.docx]

S8 Table: Disease-specific concentration index in Nepal 1995 - 2010

| Illness or symptoms | Concentration index (95% CI) | |
| --- | --- | --- |
|  | 1995 | 2010 |
| **Chronic** | -0.01 (-0.13 - 0.10) | 0.12 (0.05 - 0.18) |
| Asthma | 0.03 (-0.16 - 0.23) | 0.19 (0.01 - 0.38) |
| Diabetes | -0.36 (-1.10 - 0.39) | -0.11 (-0.28 - 0.06) |
| Heart conditions | -0.10 (-0.33 - 0.14) | 0.19 (0.04 - 0.34) |
| Epilepsy | 0.03 (-0.59 - 0.65) | -0.18 (-0.64 - 0.28) |
| Occupational illness | 0.22 (-0.15 - 0.58) | NA |
| Cancer | -0.14 (-0.62 - 0.33) | 0.09 (-0.63 – 0.81) |
| Gastrointestinal diseases | - | 0.11 (-0.03 - 0.25) |
| Rheumatism related | - | 0.13 (-0.06 - 0.32) |
| High/low blood pressure | - | 0.04 (-0.26 - 0.34) |
| Gynecological problems | - | 0.13 (-0.07 - 0.34) |
| Kidney/liver diseases | - | 0.29 (0.10 - 0.47) |
| Cirrhosis of liver | 0.24 (-0.21 – 0.69) | - |
| **Recent acute illnesses** | -0.07 (-0.13 - -0.01) | -0.17 (-0.22 - -0.12) |
| Non-specific fever | -0.12 (-0.20 - -0.04) | -0.13 (-0.22 - -0.03) |
| Diarrhea | -0.02 (-0.20 - 0.15) | -0.21 (-0.34 - -0.09) |
| Respiratory | 0.05 (-0.10 - 0.19) | -0.03 (-0.19 - 0.13) |
| Skin disease | -0.04 (-0.30 - 0.21) | -0.10 (-0.42 - 0.21) |
| Dysentery | -0.19 (-0.49 - 0.11) | 0.13 (-0.24 - 0.51) |
| Malaria | -0.12 (-0.46 - 0.22) | -0.08 (-0.24 - 0.09) |
| Jaundice | 0.49 (-0.63 - 1.61) | 0.04 (-0.24 - 0.32) |
| Parasites | 0.04 (-0.37 - 0.45) | 0.01 (-0.54 - 0.57) |
| Measles | 0.75 (-0.36 – 1.85) | NA |
| Tuberculosis | -0.06 (-0.38 - 0.25) | -0.43 (-1.96 - 1.10) |
| Cold/fever/flu | - | -0.30 (-0.41 - -0.18) |
| Dental problems | - | 0.03 (-0.40 – 0.46) |
| **Injury** | 0.01 (-0.18 - 0.21) | 0.06 (-0.07 - 0.18) |
| **Other** | -0.05 (-0.11 - 0.13) | 0.04 (-0.02 – 0.10) |

95% CI: 95% Confidence interval, NA: Not applicable
