# Supplementary material for: Cost and economic burden of illness over 15 years in Nepal: A comparative analysis
Source: PLoS One. 2018 Apr 4;13(4):e0194564. doi: 10.1371/journal.pone.0194564 (PMC5884500; doi:10.1371/journal.pone.0194564)
Supplement: S1 Fig — (DOCX) [file pone.0194564.s011.docx]

S1 Fig. Share of household consumption in Nepal, 1995 - 2010
