# Supplementary material for: Cost and economic burden of illness over 15 years in Nepal: A comparative analysis
Source: PLoS One. 2018 Apr 4;13(4):e0194564. doi: 10.1371/journal.pone.0194564 (PMC5884500; doi:10.1371/journal.pone.0194564)
Supplement: S2 Fig — (DOCX) [file pone.0194564.s012.docx]

S2 Fig. Concentration curve of household catastrophic health payment by survey year 1995 and 2010 in Nepal
